# Supplementary material for: A systematic review protocol: Efficacy and safety of nitrous oxide in analgesia in burn patients with dressing change
Source: PLoS One. 2026 Apr 17;21(4):e0347524. doi: 10.1371/journal.pone.0347524 (PMC13089730; doi:10.1371/journal.pone.0347524)
Supplement: Attachment — Submitted filename: PubMed Strategies.pdf [file pone.0347524.s004.pdf]

PubMed Strategies

#1	Search: “Nitrous Oxide”[Mesh]
#2	Search: “laughing gas”[Title/Abstract]
#3	Search: “dinitrogen oxide”[Title/Abstract]
#4	Search: “N2O”[Title/Abstract]
#5	#1 OR #2 OR #3 OR #4
#6	Search: “Burns”[Mesh]
#7	Search: “burn*”[Title/Abstract]
#8	Search: “burn injur*”[Title/Abstract]
#9	Search: “burn wound*”[Title/Abstract]
#10	Search: “scald”[Title/Abstract]
#11	#6 OR #7 OR #8 OR #9 OR #10
#12	Search: “dressing change*”[Title/Abstract]
#13	Search: “wound dress*”[Title/Abstract]
#14	Search: “bandage change*”[Title/Abstract]
#15	#12 OR #13 OR #14
#16	Search: “Analgesia”[Mesh]
#17	Search: “pain relie*”[Title/Abstract]
#18	Search: “pain manag*”[Title/Abstract]
#19	Search: “analgesic effect*”[Title/Abstract]
#20	Search: “Pain”[Mesh]
#21	Search: “ache*”[Title/Abstract]
#22	Search: “soreness”[Title/Abstract]
#23	Search: “discomfort”[Title/Abstract]
#24	#16 OR #17 OR #18 OR #19 OR #20 OR #21 OR #22 OR #23
