# Supplementary material for: A systematic review protocol: Efficacy and safety of nitrous oxide in analgesia in burn patients with dressing change
Source: PLoS One. 2026 Apr 17;21(4):e0347524. doi: 10.1371/journal.pone.0347524 (PMC13089730; doi:10.1371/journal.pone.0347524)
Supplement: Attachment — Submitted filename: Response to Reviewers.docx [file pone.0347524.s002.docx]

**To editor:**

Thank you very much for your help! We have made corrections as per your request.Sorry I didn't specifically indicate that the uploaded image is not supplementary figures, it was uploaded as an image file, the legend is on line 209.Please feel free to contact me if you still have questions. I wish you all the best!

**Reviewer 1:**

Thank you very much for your careful review!We have made the following corrections to your problem.

1. After careful consideration, we decided to strictly limit the inclusion of RCTs, which can improve the quality of evidence.

2. The reason why we only include adults is that the pain scores of children's burns are mainly assessed by medical staff through facial expressions and leg movements (FLACC scale), while adults mostly use VAS or NRS scales for self-evaluation. Different evaluation subjects may lead to heterogeneity. But you're right, it's true that burns in children are more common, and we can do subgroup analysis of children, so we made a modification in the text to include patients of all ages and do subgroup analysis according to age. The word "first time" was also clarified.

3. Different scales cannot be directly compared. We will use standardized mean differences for meta-analysis.

4. We did overlook this point, and we have added content to the "Comparators" section and the "Subgroup analysis" section.

5. You are very rigorous and the issues you point out are very important. We have added an evaluation method for non-English articles in the Screening section.

6. We corrected this error in the "Data analysis" section. Different scales do use standardized mean differences. We have added the solution to the problem of multiple measurements in the "Data extraction and records" section.

7. We agree that p < 0.05 is a more conventional threshold for hypothesis testing, and this error has been corrected.

I wish you all the best!
