# Supplementary material for: A systematic review protocol: Efficacy and safety of nitrous oxide in analgesia in burn patients with dressing change
Source: PLoS One. 2026 Apr 17;21(4):e0347524. doi: 10.1371/journal.pone.0347524 (PMC13089730; doi:10.1371/journal.pone.0347524)
Supplement: Attachment — Submitted filename: Response_to_Reviewers_auresp_2.docx [file pone.0347524.s003.docx]

**To editor:**

Thank you very much for your help!Please feel free to contact me if you still have questions. I wish you all the best!

**Reviewer 1:**

Thank you very much for your careful review!We have made the following corrections to your problem.

1. We have listed more synonyms and revised the search strategy.

2. We did not list the study design in the search strategy to prevent the omission of eligible studies. We will limit the type of study during manual screening.

3. We have optimized the search strategy according to your requirements in the table.

4. We have modified the retrieval strategy according to your example.

According to your comments on articles 5, 6, 7 and 9, we have revised the text.

8. We have made modifications in the Data extraction and records section of the article.

PRISMA Checklist We have uploaded as other files.

I wish you all the best!
